# Androgens, Endometriosis and Pain

**DOI:** 10.3389/frph.2021.792920

**Published:** 2021-12-03

**Authors:** Susan F. Evans, M. Louise Hull, Mark R. Hutchinson, Paul E. Rolan

**Affiliations:** ^1^Adelaide Medical School, University of Adelaide, Adelaide, SA, Australia; ^2^Robinson Research Institute, School of Pediatrics and Reproductive Health, University of Adelaide, Adelaide, SA, Australia; ^3^ARC Centre of Excellence for Nanoscale Biophotonics, University of Adelaide, Adelaide, SA, Australia

**Keywords:** androgen, endometriosis, chronic pain, aromatase inhibitor, hormonal, women, testosterone, immune

## Abstract

The intriguing relationship between androgens, endometriosis and chronic pain continues to unfold. Determining this relationship is of crucial importance to gynecologists managing people with these conditions, as common treatments dramatically alter her hormonal profiles, with both intended and unintended consequences. Although they may be present in the same individual, there is a recognized disconnect between pain or pain-related symptoms, and the presence or extent of endometriosis lesions. Reduced androgen levels provide a potential mechanism to link the development of endometriosis lesions and the presence of chronic pain. This research paper expands the presentation of our research at the World Endometriosis Congress in 2021, subsequently published in the Journal of Pain Research which demonstrated a strong inverse relationship between androgen levels and days per month of pelvic and period pain. Here we extend and further explore the evidence for a role for androgens in the etiology and management of dysmenorrhea and pelvic pain in women, both with and without endometriosis. We explore the potential for inflammation to induce low androgen levels and consider ways in which clinicians can optimize levels of androgens when treating women with these conditions. This article prompts the question: Is it estrogens that predispose people to a life of pain, or androgens that are protective?

## Introduction

Women are over-represented in chronic pain populations when compared to males (1, 2). Over recent years, there have been extensive and often conflicting outcomes from research seeking to determine the ways in which a female's relatively higher levels of estrogen when compared to males may predispose her to chronic pain (1, 3, 4). In contrast, research to determine the effect of androgens on pain in women is sparse. In a group of healthy women, Bartley and Fillingim (1) found higher testosterone levels to be anti-nociceptive, and higher estrogen levels to be mildly nociceptive. To our knowledge, our research is the first to document an inverse correlation between levels of testosterone and the experience of pain in young women (5). This article outlines our recent research findings and how androgens can influence pain at different stages of life and when different pathologies are present. Finally, we explore the potential for androgens to provide a therapeutic benefit in women and what role they may have in future treatment pathways.

## Research Findings

Our work, as published in the Journal of Pain Medicine (5), investigated the relationship between serum levels of 10 steroid hormones and the subjective experience of dysmenorrhea-related pelvic pain symptoms (5). We used high sensitivity liquid chromatography mass spectrometry (LC-MS) assays to measure serum levels at 2 stages of a single menstrual cycle, Day 1–2 (when estrogen is baseline) and Day 7–10 (when estrogen peaks). We measured estrogen, progesterone and androgen levels and determined their correlation with the Days per Month of Pelvic Pain (DPelvicPM), Period Pain (DPeriodPM) and Headache (DHeadachePM).

Our results showed that in non-users of the oral contraceptive (OC), there was a strong inverse correlation between a reducing Free Androgen Index (FAI) (6) and increasing DPelvicPM (*p* = 0.0032) and DPeriodPM (*p* = 0.013) on Day 1–2 (Table 1). Non-users of the OC also demonstrated a strong inverse correlation between a reducing FAI and an increasing DPelvicPM (*p* = 0.058) and DPeriodPM (*p* = 0.029) on Day 7–10. A weakly positive correlation between estradiol and DPelvicPM (*p* = 0.49) was found only on Day 7–10 in women who used the OC.

**Table 1:** Comparison of significance (*p*-value) between androgen and estrogen levels and Days per Month of Pelvic Pain (DPelvicPM), Period Pain (DPeriodPM) and Headache (DHeadachePM) on Day 1–2 and Day 7–10 of the menstrual cycle in women not using the contraceptive pill.

	**Day 1–2**			**DAY 7–10**		
	**DPelvicPM**	**DPeriodPM**	**DHeadachePM**	**DPelvicPM**	**DPeriodPM**	**DHeadachePM**
Testosterone (units)	0.17	0.24	0.13	0.99	0.85	0.54
Androstenedione	0.036^*^	0.035^*^	0.26	0.52	0.45	0.39
DHEA	0.0018^**^	0.013^*^	0.14	0.098	0.33	0.077
Free androgen index	0.0032^**^	0.013^*^	0.20	0.0058^**^	0.029^*^	0.33
Estradiol	0.30	0.37	0.61	0.068	0.20	0.78
Free estrogen index	0.50	0.98	0.88	0.71	0.74	0.92
Cortisol	0.36	0.72	0.87	0.47	0.72	0.71

* *p < 0.05*,

** *p < 0.01*.

*Figures with p < 0.05 shown in bold. The Relationship Between Androgens and Days per Month of Period Pain, Pelvic Pain, Headache, and TLR4 Responsiveness of Peripheral Blood Mononuclear Cells in Young Women with Dysmenorrhoea. Evans et al. (5). Journal of Pain Research 2021:14 585-99*.

## Androgens, Pain and Pubertal Development

Gender disparity for chronic pain is well documented, with women being over-represented in chronic pain populations (1, 2). This disadvantage begins at puberty and persists throughout a woman's life. Perquin et al. (7) studied the prevalence of chronic pain in 5,336 Dutch children aged 4–18 years. Before puberty the prevalence of chronic pain conditions in their study was approximately equal between boys and girls. However, by the age of 12–14 years girls were over-represented, and by the age of 16–18 years girls were four times more likely to report a chronic pain condition than boys. The most frequently reported chronic pain phenotype was a combination of abdominal pain and headache in a 16 to18 year old girl (7).

Both males and females produce androgens and estrogens, although in varying proportions. Senefeld et al. (8) examined the change in testosterone levels across 2,293 males and 2,202 females from ages 6 to 20 years. Between the ages of 6 and 10 years, testosterone levels were similar regardless of sex. A divergence in levels began at age 11, when males recorded higher androgen levels and females lower androgen levels. By the age of 12, the age at which Perquin noted a gender-based divergence in the prevalence of chronic pain (7), there was no overlap in the interquartile ranges between males and females, and at age 20, the average testosterone levels were 516 ng/dL (17.9 nmol/L) in males and 29.5 ng/dl (1.02 nmol/L) in females, a ratio of over 10 times.

In males, a shorter anogenital length has been shown to correlate with lower levels of androgen or exposure to endocrine disruptors such as phthalates during fetal development, and a higher rate of male reproductive disorders in later life, such as reduced testicular size and sperm count (9). Lee et al. (10) investigated 8,336 adult males and found that males with levels of testosterone < 3.5 ng/mL had significantly higher rates of chronic pelvic pain than males with levels of testosterone more than 3.5ng/mL (*p* = 0.001).

In females, a shorter anogenital length, representing lower levels of androgens during fetal development, has been correlated with a higher prevalence of endometriosis (11). A longer anogenital length in females, representing higher levels of androgens during fetal development, has been associated with an increased prevalence of Polycystic Ovarian Syndrome (PCOS) (12). Dinsdale proposed that endometriosis and PCOS represent opposite extremes of a continuum of reproductive traits, which are related, at least in part, to levels of prenatal testosterone exposure (12). However, additional factors will influence whether endometriosis or PCOS, or both conditions are expressed within an individual. Our research, showing an inverse relationship between androgen activity, and the DPelvicPM and DPeriodPM (5), is consistent with a role for androgens in females as a pain modulator.

Rates of chronic pain among transgender populations during hormonal transition offer a unique opportunity to determine the effect of changing reproductive hormonal profiles on pain. Aloisi (13) found that 55% of 26 transgender males with chronic pain reported a reduction of pain after testosterone treatment during female to male transition, whereas none reported increased pain. In contrast, 23% of 47 transgender females reported initiation of chronic pain after estrogen and anti-androgen therapy during male to female transition, and another 18% reported a greater sensitivity to pain. However, Grimstad (14) reported the pelvic pain experiences of transgender men who completed an online survey circulated through social media. Novel abdominal pain was reported by 69% of 183 transgender males who chose to respond to the survey after starting androgen treatments, with a median interval from testosterone initiation to pain onset of 1 year.

## Testosterone as an Inhibitor of Inflammation

The ability of testosterone to reduce inflammation and reduce symptoms has been established in trials in males and females with Rheumatoid Arthritis (RA) (15–17). RA is an autoimmune inflammatory condition, more prevalent in females than males, which is associated with elevated levels of the pro-inflammatory cytokine IL-1β (18). In the majority of studies in men, low levels of testosterone were associated with an increase in IL-1β, IL-6, and TNF-α, a reduction in the anti-inflammatory cytokine IL-10, and an increase in body fat mass (19, 20). Treatment with androgens inhibited IL-1β secretion by peripheral blood mononuclear cells and tissue macrophages within the synovium (21, 22), and was associated with an improvement in RA symptoms. Malkin et al. (23) found that while males with RA exhibited lower levels of testosterone in serum and synovial fluid than unaffected males, their testosterone levels prior to the onset of RA symptoms were similar to those of unaffected males. They proposed that low androgen levels are the result of inflammation, rather than an initiating cause of inflammation.

In Multiple Sclerosis, testosterone therapy is associated with a reduced expression of inflammatory cytokines, and has neuroprotective effects (24). Kanda et al. (25) reported the ability of testosterone to suppress levels of IgG anti-dsDNA in patients with Systemic Lupus Erythematosis, implying an effect of testosterone on B-cell immune function. In women with endometriosis and infertility, there is evidence for both an increase in inflammation (26), and a reduction in testosterone (27, 28).

**Figure 1:**
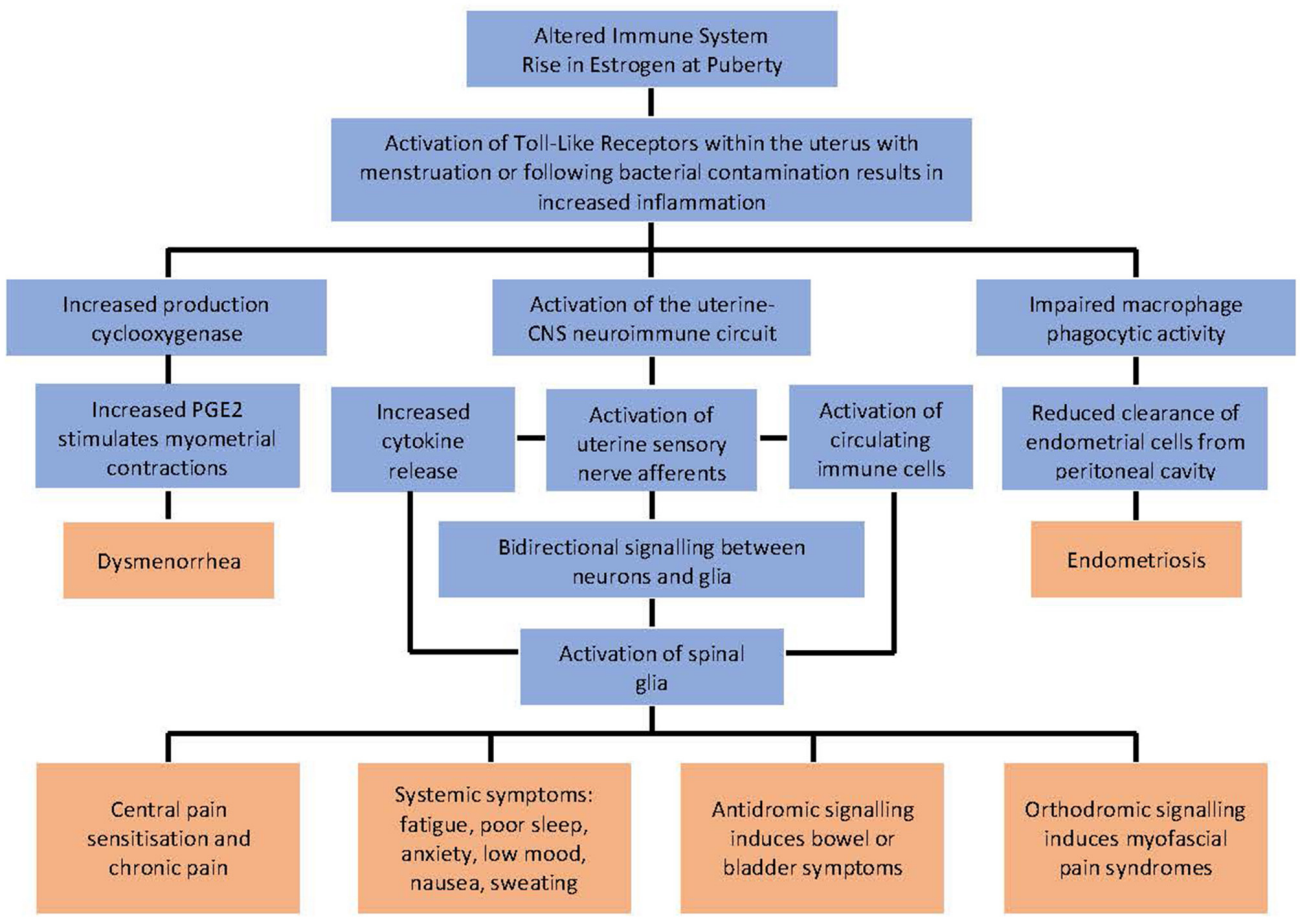
The relationship between inflammation, dysmenorrhea, endometriosis, the uterine-CNS neuroimmune circuit and their associated symptoms. Adapted with permission from Evans (29). *Dissertation. Investigations into the lived experience and etiology of dysmenorrhoea and pelvic pain in young women*.

Our group has proposed an entirely novel hypothesis (29) (Figure 1) to explain the relationship between dysmenorrhea, chronic pain and endometriosis and inflammation. This theory proposes that these three conditions develop through independent mechanisms, which may all be exacerbated by the presence of inflammation. Dysmenorrhea occurs when prostaglandin levels within the uterus rise following progesterone withdrawal with resolution of the corpus luteum. Where inflammation is present, cyclooxygenase activity is enhanced, and prostaglandin formation is increased further. Endometriosis lesions develop following retrograde menstruation. Where inflammation is present, macrophage phagocytic activity is impaired and the clearance of endometrial cells from the peritoneal cavity is reduced, facilitating the development of lesions. Symptoms including chronic pain, fatigue, poor sleep, anxiety, low mood, nausea, sweating, bowel or bladder dysfunction and myofascial pain syndromes are due to excess activation of the uterine-central nervous system neuroimmune circuit. This circuit includes the sensory afferent innervation of the uterus, circulating immune cells, circulating cytokines, and immune competent cells within the central nervous system. Inflammation results in excess activation of the neuroimmune cells within the circuit and an increase in symptoms. Antidromic neural signaling induces bowel or bladder symptoms. Orthodromic neural signaling induces myofascial pain syndromes.

This theory easily accepts the variable presentations observed in clinical practice, the range of symptoms both within and outside the pelvis that women present with, the lack of correlation between the presence or extent of endometriosis lesions and the presence or severity of symptoms (30), and the persistence of symptoms, in women following hysterectomy and endometriosis excision. Factors associated with increased inflammation, such as reduced levels of testosterone, prenatally or postnatally, enhance one or more of these processes and thus impact an individual's clinical presentation.

## Androgens and Endometriosis

Endometriosis lesions are estrogen-dependent. However, even where circulating estrogen levels are suppressed, multiple factors modify the hormonal profile within endometriosis lesions, maintaining a stimulus for growth. Delvoux et al. (31, 32) described an increased expression of the enzyme 17β-hydroxysteroid dehydrogenase Type 1 (17β-HSD Type 1) within endometriosis lesions, compared with eutopic endometrium. This favors the production of the more potent 17β-estradiol (E2) over its less potent metabolite, which displays weaker ER agonist activity. Zeitoun et al. (33) demonstrated the increased expression of aromatase within endometriosis lesions. Aromatase favors the conversion of androgens to estrogens, and importantly is found in endometriosis lesions, but not in eutopic endometrium. This combination of increased aromatase and reduced 17β-HSD within endometriosis cells results in both a higher ratio of estradiol to estrone within lesions, and a higher level of estradiol within lesions than within the circulation. Endometriosis cells have a proliferative response to these potent high estradiol levels, with promotion of lesion growth and development.

Like estrogens, androgen receptors display distinct subtypes. Classical or genomic AR activity is mediated through intracellular proteins encoded by the nuclear receptor subfamily NR3, with the single androgen receptor gene *NR3C4* located on the X-chromosome. Androgens within the cytoplasm are transported to the nucleus to bind to the AR and regulate target gene transcription. Additional non-DNA binding-dependent androgen receptors allow more rapid action, but are relatively poorly studied when compared to the non-genomic receptors that bind estrogens (34, 35). Endometriosis lesions exhibit estrogen (36), progesterone (37) and androgen receptors (38), with variable activity across the menstrual cycle.

## Potential Clinical Use of Androgens in Women

Females produce androgens in the thecal cells of the ovary in response to luteinising hormone, as well as in the adrenal gland and adipose tissue. Multiple factors including aging, oophorectomy, hypothalamic amenorrhea, premature ovarian failure, or hypopituitarism may all reduce ovarian androgen production. However, more commonly, menstrual suppression by medications that inhibit ovulation to treat dysmenorrhea leads to the unintended collateral effect of dramatically reducing androgens in young females.

Synthetic estrogens, such as ethinylestradiol in the OC, further reduce androgen activity by increasing the production of SHBG within the liver. In our research, average SHBG levels on Day 1–2 of the menstrual cycle were 92.4 in non-OC users of the OC compared to 159 in OC users (*p* = 0.0001^**^). Our research used the FAI to adjust for variable levels of SHBG among participants. On Day 1–2, the FAI was 0.87 in non-OC users compared to 0.53 in OC users (*p* = 0.0013^**^). This approach is supported by Keevil (6), who determined that use of the FAI was reliable for women of normal weight, where the SHBG was >30 nmol/l. In males, or women who are obese, with insulin resistance, or where the SHBG is < 30 nmol/l, measurement of the Calculated Free Testosterone is preferred. All participants in our research were aged 16–35 years, with regular menstrual cycles, and a body mass index of between 16 and 30. As such, the measurement of the FAI rather than the Calculated Free Testosterone was appropriate.

In view of the consistent findings that lower levels of androgens are associated with increased pain, clinicians may look to ways to maintain or enhance androgens levels in patients. Choosing an intrauterine device for contraception or menstrual suppression rather than an OC allows her to maintain ovarian androgen production. Choosing a progestogen-only systemic steroid rather than a combined OC avoids the estrogen-induced increase in liver production of SHBG, while allowing effective menstrual suppression. Progestins can mimic, inhibit or potentiate androgens (39), and the optimum progestogen for the maintenance of androgens has yet to be determined. A reduction in body mass reduces the potential for additional conversion of androgens to estrogen within adipose tissue.

Where optimizing the balance between endogenous estrogens and androgens is insufficient to improve well-being and reduce pain, the therapeutic use of testosterone at pharmacological rather than physiological levels offers an alternative but under-researched approach. Testosterone has been prescribed effectively in pharmacological doses to treat a proportion of women with fibromyalgia (40–42), anxiety (43), multiple sclerosis (24, 44), hot flushes (45), opioid-induced androgen deficiency (46), migraine headaches (47), breast cancer (48), and rheumatoid arthritis (16). The improvement in pain symptoms for fibromyalgia noted by White et al. (41) is consistent with the study by Schertzinger et al. (49), who measured levels of testosterone in 8 women with fibromyalgia each day for 25 consecutive days. Daily testosterone levels (*p* = 0.015) were significantly and inversely correlated with daily pain severity, while no relationship between estradiol (*p* = 0.551) and daily pain was found. However, pharmacological doses of androgens carry a substantial risk of virilising adverse effects in women. The development of Selective Androgen Receptor Modulators (SARMs) offers the potential for products optimized for use in women.

## Combined Testosterone and Aromatase Inhibitor Therapy

Individuals with medical conditions associated with enhanced aromatase activity, such as endometriosis or rheumatoid arthritis, require particular care where consideration is given to the clinical use of androgen therapy. This is because there is potential for the conversion of testosterone to estradiol within endometriosis lesions or synovium. Aromatase inhibitors, such as anastrozole or letrozole, prevent the conversion of testosterone to estradiol. Their ability to reduce pain and to reduce the size of endometriosis lesions has been demonstrated (50–52), but their use has been limited by adverse effects such as hot flushes, vaginal atrophy and reduced bone density. The co-administration of testosterone and an aromatase inhibitor offers potential for women with endometriosis to receive the benefit of an aromatase inhibitor while maintaining personal well-being and bone density.

## Conclusion

There has been a dearth of research into the role of androgens in the development of endometriosis lesions, the experience of pain, inflammatory disease processes, and optimizing a woman's quality of life. Our research indicates that inflammation, pain and androgen levels are intimately associated, with a lack of clarity regarding whether low androgen levels pre- or post-date the presence of inflammation. Optimal products for the treatment of testosterone deficiency in women are yet to be developed, and the potentially pharmacological doses required to achieve therapeutic benefit using existing testosterone therapies are yet to be established. Hormonal therapy for menstrual suppression profoundly influences androgen levels and may contribute to reduced androgen effect. The development of new therapies to reduce inflammation may offer a novel approach to addressing androgen deficiency in women.

## Author Contributions

All authors listed have made a substantial, direct, and intellectual contribution to the work and approved it for publication.

## Funding

This study was part funded by the Australia New Zealand College of Anaesthetists (ANZCA) Research Foundation (Grant 362/50114572) and the Australian Research Council (FT180100565).

## Conflict of Interest

SE receives royalties from book authorship, is a shareholder in Alyra Biotech Pty Ltd a company developing non-hormonal immune therapies for pelvic pain and Havah Therapeutics Pty Ltd a company developing testosterone therapies for women with breast cancer; and has patents pending: PCT/AU2018/051383 and PCT/AU2020/050551, Alyra Biotech Pty Ltd. PR is a shareholder in Havah Therapeutics, Alyra Biotech, Lipotek and iX Biopharma, a consultant to Bionomics and Novartis, and has received payment for educational presentations from Novartis and Seqirus. MRH is Director of the Australian Research Council Centre of Excellence for Nanoscale BioPhotonics CE140100003 and the recipient of an ARC Future Fellowship FT180100565, and reports grants from Australian Research Council, National Health and Medical Research Council, Meat and Livestock Australia, Air Force Office of Scientific Research, Defence Science Technology Group, and National Institutes of Health. His research program is supported by Novartis, Abbott, Pfizer and Regeneus, but these activities fall outside the submitted work. The remaining author declares that the research was conducted in the absence of any commercial or financial relationships that could be construed as a potential conflict of interest.

## Publisher's Note

All claims expressed in this article are solely those of the authors and do not necessarily represent those of their affiliated organizations, or those of the publisher, the editors and the reviewers. Any product that may be evaluated in this article, or claim that may be made by its manufacturer, is not guaranteed or endorsed by the publisher.
